# Level of knowledge, attitude, and practice on modern contraceptive method and its associated factors among housemaids living in Debre Tabor town, northwest Ethiopia: a community-based cross-sectional study

**DOI:** 10.1186/s12905-023-02783-5

**Published:** 2023-11-27

**Authors:** Gebrehiwot Ayalew Tiruneh, Besfat Berihun Erega, Awgichew Behaile T/mariam, Endeshaw Chekol Abebe, Teklie Mengie Ayele, Nega Dagnaw Baye, Zelalem Tilahun, Alebachew Taye, Bekalu Getnet Kassa

**Affiliations:** 1 Department of Midwifery, College of Health Sciences, Debre Tabor University, Debre Tabor, Ethiopia; 2 Department of Biomedical Sciences, College of Health Sciences, Debre Tabor University, Debre Tabor, Ethiopia; 3 Department of pharmacy, College of Health Sciences, Debre Tabor University, Debre Tabor, Ethiopia; 4 Department of statistics, College of natural and computational Sciences, Debre Tabor University, Debre Tabor, Ethiopia

**Keywords:** Attitude, Ethiopia, Housemaid, Knowledge, Modern contraceptive, Practices

## Abstract

**Background:**

Contraception is widely recognized as an effective technique for avoiding unplanned pregnancies and sexually transmitted diseases. Promoting contemporary contraceptive methods would minimize the number of unplanned pregnancies and the high number of maternal fatalities connected with unsafe abortions.

**Objective:**

This study aims to assess the level of knowledge, attitude, and practice of modern contraceptive methods and its associated factors among housemaid residents of Debre Tabor Town, northwest Ethiopia:

**Methods:**

A structured questionnaire supplemented with face-to-face interviews was used to conduct a community-based cross-sectional study with 423 housemaids’ women of reproductive age in Debre Tabor City. The data were analyzed using descriptive analysis, binary analysis, and multivariable logistic regression.

**Results:**

A 12.8% of respondents in this study used modern contraceptive methods. A 44.68% of study participants had good knowledge of modern contraceptive methods, and 36.40% had a positive attitude towards them. Housemaids’ older age, urban location, educational status, work experience, and family situation were found to be positive predictors of a good understanding of current contraceptive techniques. Housemaids’ older age, urban residence, educational level, work experience, family situation, and first sex before now are all positive predictors of a positive attitude and good practices.

**Conclusions:**

Housemaids’ knowledge, attitude, and practice of modern contraceptive methods were influenced by a variety of socio-demographic factors. As a result, housemaids should be educated about modern contraceptive methods by the health sector and other stakeholders to improve their knowledge, attitude, and practices.

## Introduction

Family planning is the use of various methods and strategies to enable men and women to make educated decisions regarding childbearing [1, 2]. It refers to the methods used by men and women to space their pregnancies and limit the number of children they plan to have [3, 4]. It encompasses the services, policies, information, attitudes, practices, and commodities, such as contraceptives that allow women, men, couples, and adolescents to avoid unintended pregnancy or decide when to have a child [5]. Family planning is concerned with the mother’s reproductive health, adequate birth spacing, avoiding unwanted pregnancies and abortions, preventing sexually transmitted diseases, and increasing the mother’s quality of life, as well as the children’s and families’ in general [2]. Short-term modern family planning methods are currently available at all levels of governmental and private healthcare units, while long-term options are offered in health centers, hospitals, and certain private clinics [5, 6].

The International Birth Spacing Policy encourages women to use reversible contraception such as oral contraceptives, depo-provera, condoms, and Intra Uterine Devices (IUDs). By expanding the spectrum of reversible and cheap contraceptives, this program promotes reproductive-age women to access the complete range of contraceptive services [7].

Contraception is regarded as an important preventive measure for unintended pregnancies and sexually transmitted diseases, such as human immunodeficiency virus infection and acquired immune deficiency syndrome (HIV/AIDS), among adolescents [8].

Unintended pregnancies, maternal and child mortality, and induced abortions are all reduced when people practise family planning. Contraception has also been shown to increase woman’s sense of autonomy and ability to make decisions in other areas of her life. Contraception could save at least 25% of all maternal deaths by preventing unplanned pregnancies and unsafe abortions, as well as sexually transmitted diseases like HIV, Chlamydia, and Syphilis. In developing countries including Ethiopia, the major barriers to family planning adoption are a lack of knowledge about contraceptive methods, their source of supply, cost, or inadequate accessibility [6, 9]. According to a study conducted in Jimma, Ethiopia, good contraceptive knowledge does not always equate with high contraceptive use. According to many studies, high contraceptive awareness but low contraceptive use makes the condition of family planning services a severe concern. It is critical to ensure that all pregnancies are wanted or intended on a global and national basis [6, 10].

In India, however, universal adoption of the small-family norm remains a pipe dream. Only approximately 54% of currently married women aged 15–49 years or their husbands used a contraceptive technique to control their fertility in 2007-08, and the contraceptive prevalence rate appears to have plateaued after 2004. Furthermore, India’s contraceptive practice is reported to be strongly skewed toward terminal 11 techniques, implying that contraception is used mostly for birth limitation rather than birth planning in the country [4, 8]. According to the World Health Organization (WHO), 94% of the world’s population resides in nations with policies that encourage the use of family planning. Despite these policies, a significant proportion of couples of reproductive ages do not use adequate fertility control strategies [9, 11].

As a result, initiatives aimed at improving reproductive health by increasing the use of modern contraceptives must expressly target adolescent females at all levels of the program. As a result males should be actively involved at the knowledge level (the concept of family planning), the supporting level (being supportive of others who use contraception), and the acceptor level (accepting contraception) as the contraceptive user. To promote contraceptive use, males’ decision-making roles should be considered [6, 10, 11]. Despite several modern contraceptives being available worldwide, the problem of unwanted births persists, which may be attributed to a lack of information and a misunderstanding of contraception. Understanding community contraceptive use is critical to understanding variations in fertility and reproductive health in various parts of the world. Acceptance of children as God’s plan, attitudes toward avoiding conception, awareness of different methods, and comprehension of the adverse effects of different methods are all characteristics linked to contraceptive use, according to previous studies [7, 11–13].

In developing countries, vulnerable and marginalized adolescents receive more research and programmatic attention. In terms of background, working habits, self-esteem and social ties, and exposure to HIV and adolescent programs, a descriptive analysis done to compare female domestic workers with other adolescent girls and boys revealed that they constituted 15% of the female adolescent population, the majority of whom had come from rural areas [9, 10, 14]. Housemaids were less likely than other adolescent groups to be educated or to live with their parents. They worked extraordinarily long hours for very little payment, with a monthly average salary of US$6. Domestic workers had worse self-esteem and fewer friends than other teens, and less HIV knowledge and engagement in existing adolescent programs. Despite their vast numbers in some urban areas, adolescent domestic workers are particularly vulnerable and usually unseen. These greater, at-risk groups of adolescent girls require more programmatic attention and awareness-raising [10, 13, 15].

However, there is limited data on female adolescent housemaids in Ethiopia, who may make up a significant fraction of urban girls in some areas. Most notably, in Debre Tabor, Ethiopia, there are no previous studies conducted among female domestic workers in the reproductive age group with the goal of determining their knowledge, attitude, and practice on family planning. Most reproductive-age women, particularly female domestic workers, have limited or erroneous understanding of family planning options. Moreover, despite the fact that some contraceptives are named, female domestic workers have no idea where to buy them or how to use them. In addition, these females have a negative attitude toward family planning, and some have heard erroneous and misleading information. Therefore, the current study aimed to analyze the knowledge, attitude, and practice (KAP) of FP among female domestic workers in the reproductive age range residing in Debre Tabor town, Ethiopia.

## Methods

### Study design and period

A community-based cross-sectional study design was employed in Debre Tabor town from April 4 to 30, 2022.

### Study setting

The research was conducted in Debre Tabor Town Administration, the capital city of the South Gondar Administrative Zone of Amhara National Regional State in Northwest Ethiopia. The town is 666 km far from Ethiopia’s capital city, Addis Ababa. The source population consisted of all housemaids’ reproduction-aged women and up living in Debre Tabor. The town is divided into six kebeles.

### Participants

All housemaid’s female found in the Debre Tabor town administration were used as the source population. While the study population was housemaids who live at least for a month during the study period. All systematically selected female housemaid’s reproductive-aged women and up who lived in the study area for at least a month during the study period were included in the study. But non-residential workers (not living together with the employer) and housemaid females who had a serious illness during the study period were excluded from the study.

### Variables

#### Dependent variables

Knowledge, Attitude and practice of modern contraceptive methods.

#### Independent variables

socio-demographic characteristics (Age of housemaid, Previous residence of housemaids, Marital status of housemaids, Educational status of housemaid, Housemaid religion, Family situation of housemaids, Frist sex before now, Salary of housemaid, Working experience, Age of female employer, Age of male employer, Employer’s marital status, Employer’s religion, Educational status of female employer’s, Educational status of male employer’s, Khat-chewing history, smoking cigarrate and alcohol consumption history); Knowledge of modern contraceptive, attitude of modern contraceptive and practice of modern contraceptive methods, health facility related factors etc.

### Operational definitions

#### Housemaid

The girl or woman who is a servant employed to do housework.

#### Knowledge of modern contraceptives methods

Thirty questions about modern contraceptive methods were asked to test the participants’ knowledge. The responses to each question were coded as “1” for “yes” and “0” for “no.“

#### Attitude towards modern contraceptive methods

Seven attitude-related questions were used to assess participants’ attitudes toward modern contraceptive methods. A likert scale was used to answer the questions.

#### Modern contraceptive practices

When a woman of reproductive age is reported using any method of modern contraceptives (e.g., injectable, regular pills, emergency pills, Implanon, intrauterine device, condom, and surgical methods) [1].

#### Good practice

Those housemaids utilized at least one in life until kwon by given questions.

### Measurement

The respondents’ knowledge was evaluated using 30 questions, and the correct answers of each respondent for all questions were added together to determine whether the respondent had poor or good knowledge. Attitude questionnaires have seven questions that can be answered yes or no. Modern contraceptive method questionnaires also include 9 yes/no questions. To determine knowledge, attitude, and practice of modern contraceptive methods, the mean value of each variable for each respondent and the overall mean was determined.

### Sample size determination

The sample size was calculated using the single population proportion formula, and the required sample size for this study was determined using the following assumptions: desired precision (d) = 5%, confidence level = 95% (Za/2 = 1.96 value), and 50% (no study conducted) of the prevalence of KAP in female housemaids.

$$\text{n}=\frac{p(1-p){z}_{\partial /2}^{2}}{{d}^{2}} = \frac{0.5(1-0.5){1.96}^{2}}{{0.05}^{2}} = 384$$

As a result, adding 10% non-response rate the minimum sample size of the study was 423.

### Sampling procedures

There are six kebeles in the study area; to start collecting data, the number of households with female housemaids in each kebele was determined using the kebele registration book. The study households were then chosen using a simple random sampling technique based on the proportion of households in each kebeles, with the first household chosen by lottery. When two or more eligible female housemaids are found in the same household, the lottery method is used to interview only one.

### Data collection instrument and procedures

Data collection tools comprised structured questionnaires that were prepared after a thorough literature review and the study area’s local situation, and the study’s purpose was considered to design of the questionnaire. Questionnaires were prepared first in English and then translated into Amharic which is the vernacular language of the respondents by language experts for ease of understanding the respondents. Data were collected via face-to-face interview techniques using structured questionnaires.

Nine BSc-educated midwives were chosen and trained for data collection and supervision, respectively. They had prior experience with data collection. Data on socio-demographics, knowledge, attitude, and practice of family planning were gathered. Before collecting final data, questionnaires were pretested on 29 (5%) women from Woreta Town in South Gondar Zone. After the pre-test, the investigators and research assistants were involved in incorporating changes to the questionnaires. Only completed questionnaires were used to ensure internal validity.

### Stastical analysis

Data were cleaned, coded, and entered into Epidata version 4.2 before being exported to SPSS version 25 for analysis. To summarize the data, a descriptive analysis was performed. A binary logistic regression analysis was performed to determine the association of predictors and outcome variables. All predictor variables with *p* ≤ 0.2 were entered into multivariable logistic regression analysis; a significant association based on *p* ≤ 0.05, and an adjusted odd ratio (AOR) with 95% CI were identified. The results were presented in the form of texts, figures and tables.

## Results

### Socio-demographic characteristics of participants

Of the study participants, almost half the study of participants, 193 (45.6%) were in the age of 19 years. 174 (58.9%) of the 423 study participants had previously lived in a rural area. About 19.9% were illiterate, while 40.7% had completed college education or higher. The average age of the participants was 24.54 (**±** 6.25 SD). About 296 housemaids (70%) were orthodox Christians. 381 (90.1%) of all participants were single housemaids. Regarding the family situation of housemaids, approximately 55 (13%) of them have both their father and mother dead, and the average salary for housemaids was 706.62(± 134.69) ETB (see Table 1).

**Table 1:** Socio-demographic characteristics of study participants in Debre-Tabor Town, northwest Ethiopia, 2022

Study variable	Categories of variable	Frequency	Percent
Age of housemaid	≤ 19	193	45.6
	20–24	152	35.9
	≥ 25	78	18.4
Previous residence of housemaids	rural	249	58.9
	urban	174	41.1
Marital status of housemaids	single	381	90.1
	Divorced	17	4.0
	Widowed	25	5.9
Educational status of housemaid	Illiterate	84	19.9
	Primary education	94	22.2
	Secondary education	73	17.3
	college education and above	172	40.7
Housemaid religion	Orthodox	296	70.0
	Muslim	40	9.5
	Others	87	20.6
Family situation of housemaids	Both alive	206	48.7
	Only father alive	83	19.6
	Only mother alive	79	18.7
*	Both dead	55	13.0
Frist sex before now	No	242	57.2
	Yes	181	42.8
Salary of housemaid	< 750	208	49.2
	750–1000	195	46.1
	> 1000	20	4.7
Working experience	< 2 years	224	53
	2-4 years	68	16.1
	> 4 years	131	31

The majority, 306 (72.3%) employers were orthodox Christians. Regarding the educational status of employers, almost 110 (26%) employers are illiterate. The majority, 274 (64.8%), of employers were married. Of all participants employers 18 (4.3%) of the employers had a smoking cigarettes, and 48 (11.3%) employers had a chewing chat (see Table 2).

**Table 2:** Socio-Demographic, Behavioral characteristics of employers in Debre-Tabor Town, northwest Ethiopia, 2022

Study variable	Categories of variable	Frequency	Percent
Age of female employer	≤ 29	55	13
	30–49	222	52.5
	≥ 50	146	34.5
Age of male employer	≤ 29	28	6.6
	30–49	217	51.3
	≥ 50	178	42.1
Employer’s marital status	Single	64	15.1
	married	274	64.8
	divoresd	42	9.9
	windoed	43	10.2
Employer’s religion	Orthodox	306	72.3
	Muslim	62	14.7
	Others	55	13.0
Educational status of female employer’s	Illiterate	110	26.0
	Primary education	92	21.7
	Secondary education	63	14.9
	college and above	158	37.4
Educational status of male employer’s	Illiterate	43	10.2
	Primary education	67	15.8
	Secondary education	52	12.3
	college and above	261	61.7
Khat-chewing history	Yes	48	11.3
	No	375	88.7
smoking cigarate	Yes	18	4.3
	No	405	95.7
Alcohol consumption history	Yes	114	27.0
	No	309	73.0

### Knowledge status of participants on modern contraceptive methods

**Fig. 1:**
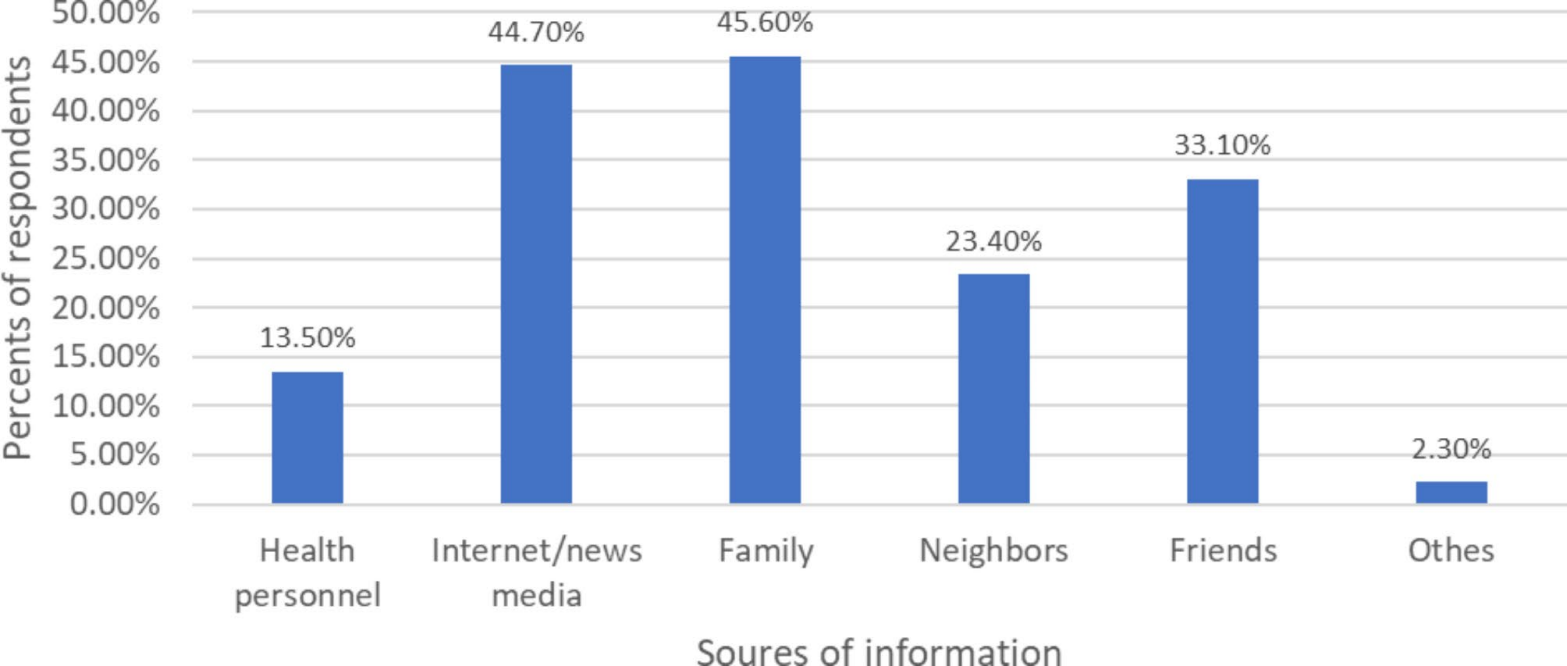
Source of information about modern contraceptive method in Debre-Tabor Town, northwest Ethiopia, 2022

**Fig. 2:**
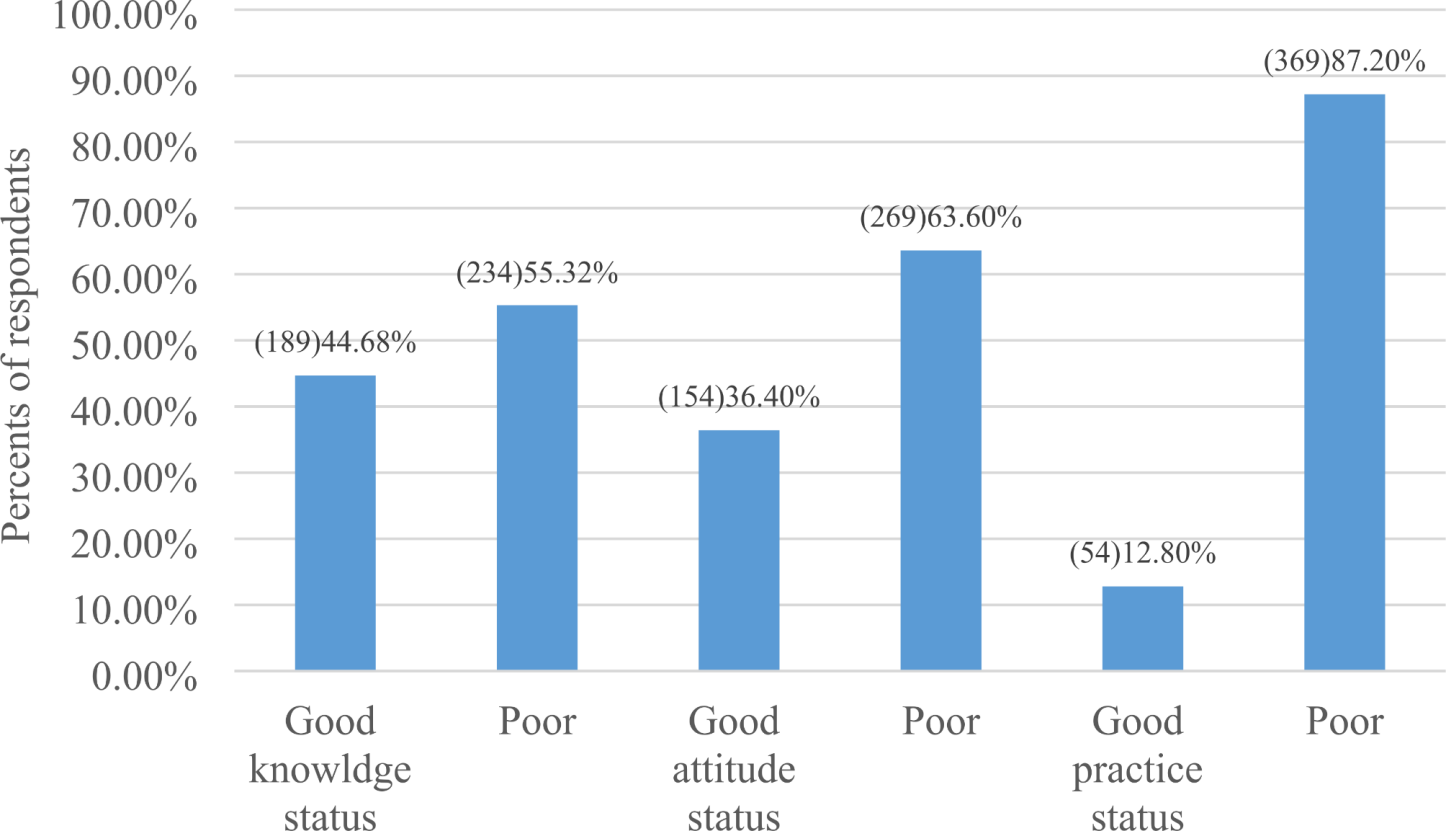
The respondents ‘level of knowledge Attitude, and Practice of Housemaids on modern contraceptive methods in Debre-Tabor Town, northwest Ethiopia, 2022

Almost half of participants ever heard about modern contraceptive methods. The major sources of information were from family (45.60%) and news media (44.7%). Among study participants knowing injectable contraceptive 53%% (see Fig. 1). Regarding the overall knowledge of study participants, 189 (44.69%) had good knowledge towards modern contraceptive methods (see Table 3) and (see Fig. 2).

**Table 3:** The respondents’ knowledge on modern contraceptive method in Debre-Tabor Town, northwest Ethiopia, 2022

Variables	Categories	Frequency	Percent
Have you ever heard about modern contraceptive method	Yes	183	43.3
	No	240	56.7
What is importance of modern contraceptive method?	Limiting of number of children	153	36.2
	spacing of birth intervals	100	23.6
	Stopping births	115	27.2
	Do not know.	55	13
Where did you get the modern contraceptive?	Private clinic	115	27.2
	Government hospital	183	43.3
	Health center	180	42.6
	Pharmacy/Drug vendor	100	23.6

### Attitude status of participants on modern contraceptive methods

Almost of the study participants 141 (33.4%) ever discussed on about contraceptive methods issues with their empowers. About 35.9% of the participants reported that they believe modern contraceptive methods exposes to infertility. Almost 192 (43.5%) of study participants reported that using modern contraceptive methods affect daily activities (see Table 4). Regarding the overall attitude, 154 (36.40%) of the participants had good attitude towards modern contraceptive methods (see Fig. 2).

**Table 4:** The respondents’ attitude on modern contraceptive method in Debre-Tabor Town, northwest Ethiopia, 2022

Variables	Agree	Disagree	Do not know
Do you believe that contraceptives us help to control birth?	(147)34.8%	(157)37.1%	(119)28.1%
Do you think that using contraceptives affect daily activities?	(184)43.5%	(219)51.8%	(20)4.7%
Do you believe that using contraceptives affect the sexual desire of the partners?	(192)45.4%	(188)44.4%	(43)10.2%
Do you believe that discussing about contraceptive methods is important?	(141)33.3%	(184)43.5	(98)23.2%
Do you think that contraceptive methods make couples infertile?	(217)51.3%	(184)43.5%	(22)5.2%
Do you think that using contraceptive methods have negative impact on practicing religion?	(158)37.4%	(226)53.4%	(39)9.2%
Do you think that contraceptive methods make couples infertile?	(152)35.9%	(253)59.8%	(18)4.3%

### Practice status of participants on modern contraceptive methods

**Fig. 3:**
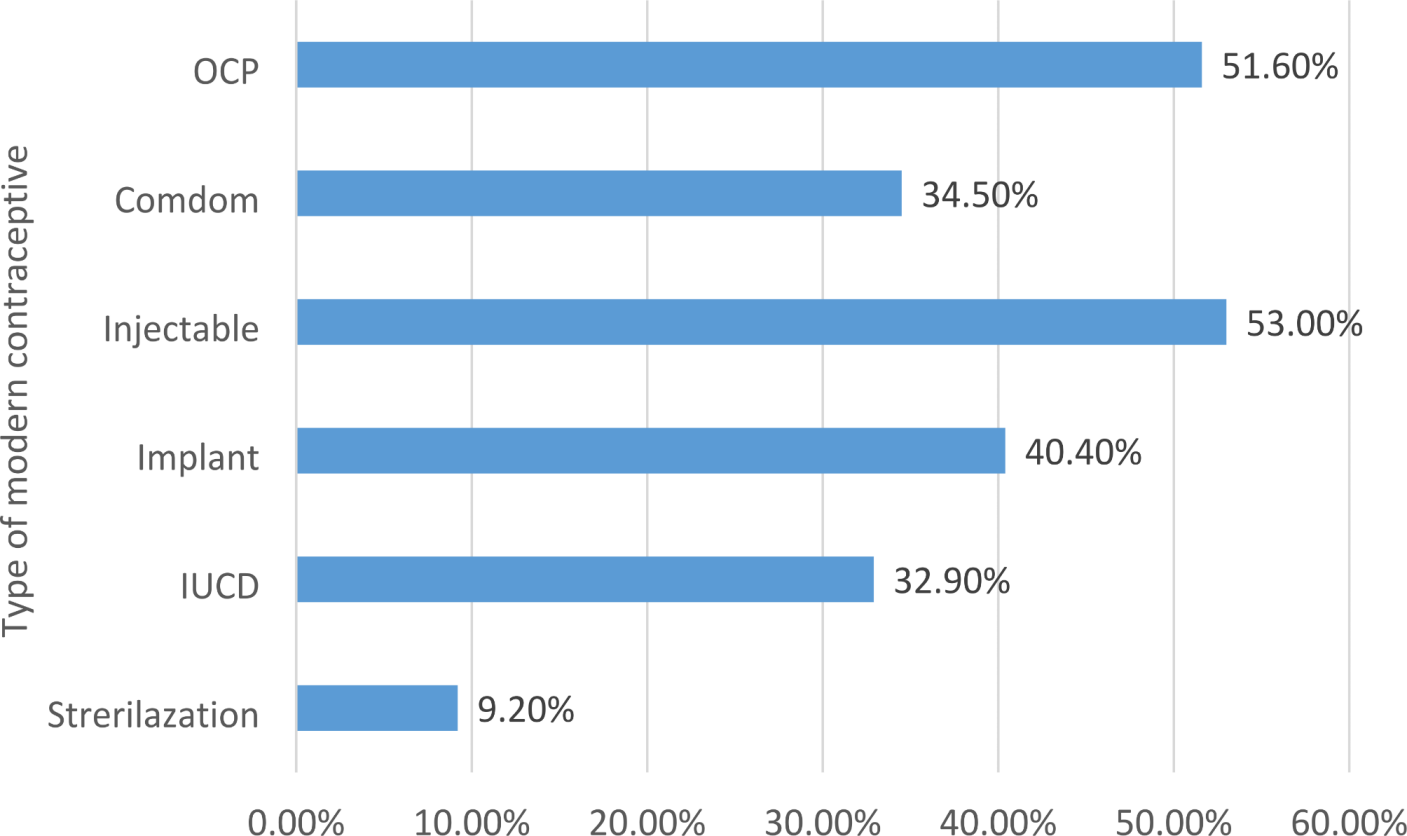
Type of modern contraceptive method in Debre-Tabor Town, northwest Ethiopia, 2022

One fourth (23.2%) of study participants ever used contraceptive methods (see Table 5). The main types were Oral contraceptive pills 47 (29.56%) and injecTable 44 (27.67) (see Fig. 3). Almost 54 (12.8%) of study participants had good practice and the rest 87.2% had poor practice (see Fig. 2).

**Table 5:** The respondents’ practice on modern contraceptive method in Debre-Tabor Town, northwest Ethiopia, 2022

Variables	Categories	Frequency	Percent
Have you ever been counseled about contraceptive methods?	Yes No	98 325	23.2 76.8
Have you ever used any of the contraceptive methods	Yes No	54	12.8
		369	87.2
Type of contraceptive did you used	Oral contraceptive pills Condoms Injectable Calendar method	47 37 44 31	29.56 23.27 27.67 19.50
Do you get contraceptive methods of your choice?	Yes	54	87.2
	No	369	12.8
Do have open discussion with your employers regarding sexual and reproductive health issues	Yes	165	39
	No	258	61
Did you encounter any side effect related with contraceptive/s utilized?	Yes	49	11.6
	No	374	88.4

### Factors associated with knowledge, attitude, and practice of housemaids on modern contraceptive methods

In the multivariable logistic regression analysis; respondent’s current age of housemaids, previous residence of housemaids, educational status of housemaids, Work experience of housemaids, the family situation of housemaids, and first sex before now, remained statistically significantly associated with housemaid knowledge, attitude, and practice of the modern contraceptive method.

Hence, the increased age of housemaids was 7. 78 times less likely to have housemaid knowledge about the use of modern contraceptive methods (AOR = 7.78; 95%CI: 4.70, 9.87). In addition, Hence, the increase housemaids’ current age was 2.19 times less likely to have a housemaid attitude to use the modern contraceptive method (AOR = 2.19; 95%CI: 2.01, 3.88**) (**see Table 6**).**

**Table 6:** Multivariable Analysis factors associated with in Debre-Tabor Town, northwest Ethiopia, 2022

Variable	Categories	Knowledge AOR(95% CI)	Attitude AOR(95% CI)	Practice AOR(95% CI)
Age of housemaid	≤ 19	1	1	1
	20–24	7.78(4.70,9.87) **	5.84 (2.77,8.92) **	1.8 (1.71,11.90) *
	≥ 25	1.80 (1.23,6.90) **	3.42 (2.08, 4.27) *	1.23(0.14, 12.01)
Previous residence of housemaids	Rural	1	1	1
	Urban	2.19 (2.01,3.88) **	9.04 (1.33,61.43) *	3.21 (1.06,6.76) *
Educational status of housemaids	Illiterate	1	1	1
	Primary education	1.24 (0.12, 13.04)	0.32 (0.07, 1.37)	1.24(0.12, 13.04)
	Secondary education	2.18 (0.26, 18.45)	0.53(0.12, 2.39)	2.18(0.26, 18.45)
	College/University	5.91 (4.76, 24.01) *	2.01(1.01,3.98) *	8.91(1.16, 28.67) *
Female employer’s educational status	Illiterate	1	1	1
	Primary education	4.70(0.13,19.60)	1.77(0.71,4.415)	0.72(0.20,2.64)
	Secondary education	0.37(0.14, 2.39)	0.70(0.75, 11.54)	0.54 (0.82,11.80)
	College/University	0.39(0.02, 1.22)	0.15 (0.23, 1.72)	0.70 (0.14,2.26)
Female Employer’s educational status	Illiterate	1	1	1
	Primary education	1.2(0.46, 3.04)	0.23 (0.28, 1.44)	1.10(0.27, 12.53)
	Secondary education	4.60 (0.13, 14.78)	0.25(0.17, 3.50)	1.41 (0.36, 2.71)
	College/University	7.23 (0.95, 30.60)	0.81 (0.29, 2.24)	0.94 (1.12, 11.25)
Employer’s marital status	Single	1	1	1
	Married	0.41(0.14,1.17)	0.66(0.37,1.20)	0.38(0.166,3.85)
Family situation of housemaids	Both alive	1.18(0.06,8.60)	1.12(1.22,3.61)	1.13 (0.02,7.61)
	Only father alive	4.13(0.04,6.43)	0.18 (0.04, 2.83)	0.18 (0.04,3.82)
	Only mother alive	3.07 (0.01, 5.44)	0.01 (0.01, 7.11)	0.54(0.25,1.14)
	Both dead	1	1	1
Work experience	< 2 years	1	1	1
	2–4 years	6.11(1.41,26.54)*	6.79(2.92,15.78) **	11.67(1.54,88.63)*
	> 4 years	2.65 (1.90, 3.69) **	1.73 (1.36, 2.19) **	2.15(1.51, 3.08) **
Frist sex before know	No	1	1	1
	Yes	1.41(1.49, 4.10)	2.69(1.17, 6.16)	7.21(2.23,23.27)
Age of female employer	≤ 29	1	1	1
	30–49	0.22(0.05,1.02)	0.98(0.38,2.55)	0.38(0.44,7.94)
	≥ 50	0.95(0.93, 2.97)	1.02(0.98, 1.06)	3.80(0.33, 20.75)
Age of male employer	≤ 29	1	1	1
	30–49	0.43(0.49, 3.98)	0.97 (0.55, 1.71)	0.08 (0.02,1.13)
	≥ 50	1.01 (0.96, 1.05)	1.09 (0.60, 1.96)	0.94 (0.93, 0.97)

1 = Reference, * *p*-value < 0.05, ** *p*-value < 0.001

## Discussions

This study was done to assess the level of knowledge, attitude, and practice on modern contraceptive methods and their associated factors among housemaids living in Debre-Tabor Town, and there was a lack of similar studies, even in other countries. Given these constraints, the findings of this study are discussed below. This study found that the prevalence of knowledge, attitude, and practice about modern contraceptive methods was low, with 44.68%, 36.4%, and 12.80%, respectively. The research revealed that a significant number of housemaids of reproductive age were not aware of contraceptive methods, unfortunately, there is very limited data and unclear understanding of the knowledge has been problematic, this was insufficient, with reported (44.68% of the total housemaids). The injectable method of modern contraception was the one with the highest awareness rate (53%) while sterilization had the lowest awareness rate (9.2%).

According to the findings of this study, as housemaids’ ages increased, so did their knowledge, attitude, and practice of modern contraceptive methods. Furthermore, urban housemaids were approximately 2.19 times more likely to have good knowledge, 9.04 times more likely to have a positive attitude, and 3.21 times more likely to practice modern contraceptive methods. The reason could be because women in urban areas are more likely to be more educated, and they have better access to information, education, and health facilities than rural women. Furthermore, the availability of major sources of family planning information, such as privet health facility and newspapers, are still limited in rural areas.

Furthermore, having a positive attitude towards modern contraceptive methods was 2.01 times more likely among housemaids with college and above educational levels compared to no education, and having practise with modern contraceptive methods was 8.91 times more likely among housemaids with college and above educational levels compared to no education. This is explained by the idea that housemaids with higher educational levels have better access to health care information, more independence to make their own and informed decisions, and a greater ability to use health care services. The positive effect of education assists housemaids in increasing their understanding of reproductive health issues as well as understanding and using the various contraceptive methods that best suit their health condition. It also improves housemaids’ overall status in terms of knowledge, attitude, and practice of modern contraceptive methods.

According to this study, knowledge, attitude, and practice of modern contraceptive methods increased with age in housemaids with work experience. Possible explanations include improved access to health-care information and increased independence to make their own informed decisions. Generally, Modern contraceptive method information is not freely available but rather flows through hidden informal or “underground” channels. Women who have previously used the method or who have been close to women in a similar situation, women’s health organizations, health professionals, pharmacies, and internet sites are the ones who provide information or help to identify sources of information, such as female relatives, friends, neighbors, and the sexual partner.

Overall, the use of contraceptive methods among housemaids of reproductive age women in this study is positive, with the majority of housemaids believing that contraceptives are beneficial.

### Strength and limitation of the study

#### Strength

This study has focused on a marginalized group of people who are highly vulnerable to unintended pregnancy due to the lack of the contraceptive method where adequate information and studies are lacking. This might certainly serve as baseline information and fill some of the knowledge gaps for further studies.

#### Limitation of the study

As the data were collected using face-to-face interview, study participants might not feel free and the reported KAP might be overestimated or underestimated. We do not used qualitative method of data collection to gather study participant’s internal feeling about modern contraceptive methods, so that triangulation was possible. In addition, barriers for utilizing contraception not addressed.

## Conclusions

A significant number of housemaids have inadequate knowledge, attitudes, and practices regarding modern contraceptive methods. Housemaids’ knowledge, attitude, and practice of modern contraceptive methods were influenced by a variety of socio-demographic factors.

As a result, housemaids should be educated about modern contraceptive methods by the health sector and other stakeholders to improve their knowledge, attitude, and practices. Increasing programmed coverage and access to modern contraceptive methods will not suffice unless all eligible women understand the importance of maintaining a positive attitude and practicing when necessary.

## Data Availability

The datasets used and/or analyzed in this study can be obtained from the corresponding author on request.
